# Development of genome-wide insertion and deletion markers for maize, based on next-generation sequencing data

**DOI:** 10.1186/s12864-015-1797-5

**Published:** 2015-08-13

**Authors:** Jian Liu, Jingtao Qu, Cong Yang, Dengguo Tang, Jingwei Li, Hai Lan, Tingzhao Rong

**Affiliations:** Maize Research Institute, Sichuan Agricultural University, Chengdu, 611130 China; Key Laboratory of Biology and Genetic Improvement of Maize in Southwest Region, Ministry of Agriculture, Sichuan Agricultural University, Chengdu, 611130 China

**Keywords:** Maize, Next-generation sequencing, Insertion and deletion, Indel, Molecular marker, Polymorphism

## Abstract

**Background:**

Insertions and deletions (indels) are the most abundant form of structural variation in all genomes. Indels have been increasingly recognized as an important source of molecular markers due to high-density occurrence, cost-effectiveness, and ease of genotyping. Coupled with developments in bioinformatics, next-generation sequencing (NGS) platforms enable the discovery of millions of indel polymorphisms by comparing the whole genome sequences of individuals within a species.

**Results:**

A total of 1,973,746 unique indels were identified in 345 maize genomes, with an overall density of 958.79 indels/Mbp, and an average allele number of 2.76, ranging from 2 to 107. There were 264,214 indels with polymorphism information content (PIC) values greater than or equal to 0.5, accounting for 13.39 % of overall indels. Of these highly polymorphic indels, we designed primer pairs for 83,481 and 29,403 indels with major allele differences (i.e. the size difference between the most and second most frequent alleles) greater than or equal to 3 and 8 bp, respectively, based on the differing resolution capabilities of gel electrophoresis. The accuracy of our indel markers was experimentally validated, and among 100 indel markers, average accuracy was approximately 90 %. In addition, we also validated the polymorphism of the indel markers. Of 100 highly polymorphic indel markers, all had polymorphisms with average PIC values of 0.54.

**Conclusions:**

The maize genome is rich in indel polymorphisms. Intriguingly, the level of polymorphism in genic regions of the maize genome was higher than that in intergenic regions. The polymorphic indel markers developed from this study may enhance the efficiency of genetic research and marker-assisted breeding in maize.

**Electronic supplementary material:**

The online version of this article (doi:10.1186/s12864-015-1797-5) contains supplementary material, which is available to authorized users.

## Background

Maize (*Zea mays* ssp. *mays*) is an incredibly important cereal crop grown widely throughout the world. Increased demand for maize owing to population growth and biofuel production, and the impacts of climate change on maize production will ratchet up the pressure for increased and more sustainable maize production. Since the 1980s, molecular markers have been widely used in maize genetics and breeding. High-density molecular markers in applied and basic research are advantageous and necessary for map-based cloning and genome-wide association study.

In contrast to single nucleotide polymorphisms (SNPs), Insertions and deletions (indels) are the second most common type of polymorphism. In a previous study, Mills reported that indels are distributed throughout the human genome at an average density of one indel per 7.2 Kb [1]. Several studies have suggested numerous indels that may cause human disease [2]. One of the most common genetic diseases in humans, cystic fibrosis, is frequently caused by various indels in coding regions of the *CFTR* gene [3]. The genetic diseases of tuberous sclerosis, Rett syndrome, and hemophilia B are also caused by small indels [4–6]. Similarly, indels can alter the phenotype of plants (e.g. the maize domestication gene *teosinte branched*; the gene *Gn1a*, which increases the number of reproductive organs in rice; and the wheat stripe rust resistance gene *Yr36* [7–9]). Consequently, indels have been increasingly recognized as an important source of molecular markers, and indel markers have been successfully used for several genetic studies in crops [10].

The maize genome was completed in 2009 using traditional Sanger sequencing technology, [11]. However, the subsequent development of next-generation sequencing (NGS) technology has generated an enormous amount of short reads that science is scrambling to analyze. NGS technology has also drastically reduced the time and cost requirements of sequencing, which has enabled the re-sequencing of a large number of genomes. This has provided for the possibility of large-scale genetic variation surveys, where many individuals within a single species have been sequenced. Examples include 40 silkworm samples [12], 31 soybean samples [13], 900 sorghum samples [14], and 1800 rice samples [15–18]. As of 2012, more than 350 maize inbred line and landrace genomes had been completely re-sequenced and published [19–21]. Rapid bioinformatics developments have introduced various software programs designed to identify indels, including Dindel [22], VarScan [23], GATK [24], and SAMtool’s mpileup [25].

The first step in most of these programs is to map reads directly to a reference sequence. Accurate indel calls from reads are challenging for a couple of reasons. First, reads covering indels are generally more difficult to map correctly to reference genomes, especially ones containing large indel events. Moreover, incorrect alignments at the nucleotide level lead to an incorrect placement of gaps in the alignments. Schuler proposed a PCR amplification computer simulation concept known as the electronic polymerase chain reaction (e-PCR), which has been used widely in various aspects of biology, including the chromosomal localization of DNA sequences, genomic sequencing, PCR primer design, and gene cloning [26]. Unlike traditional methods of sequence alignment, e-PCR programs search sequence databases using fragments similar in length to actual PCR primers that would target both ends of a sequence, instead of using a full-length sequence. Coupled with these developments in bioinformatics, NGS data enables the discovery of genome-wide indel polymorphisms by comparing the whole genome sequences of individuals within a species. Lai et al. re-sequenced a group of six elite maize inbred lines, and uncovered more than 30,000 polymorphic indels with sizes smaller than six bp [19]. Despite such progresses, information regarding indel polymorphism, size, and alleles is still inadequate, particularly considering the indel polymorphism levels observed in large populations.

We developed a set of highly polymorphic indel markers with large size differences and high-density occurrence using the NGS reads of 344 maize inbred lines and landraces along with one B73 maize reference genome [11], which was used as the template. Furthermore, we identified and analyzed these genome-wide indel polymorphisms among the populations employed using an e-PCR strategy, with the aim of enhancing the efficiency of maize genetic research and molecular marker assisted breeding.

## Results

### Identification and distribution of unique primers in the maize genome

We designed a total of 102,929,122 pairs of e-PCR primers using the maize B73 genome as a template. Of these, 93,492,302 pairs are located in intergenic regions, and 9,436,820 pairs are located in genic region, accounting for 90.83 % and 9.17 % of the total, respectively (Table 1). We mapped 11,807,240 of these primer pairs to unique genomic regions, 11.47 % of the total. Of these, 7,569,844 pairs are located in intergenic regions, and 4,237,396 pairs are located in genic regions, accounting for 64.11 % and 35.89 % of the unique primer pairs, respectively. Chromosome 1 contains the maximum number of unique primers (1,925,944), whereas chromosome 10 has the least (861,222). This implies that the number of primers located on a particular chromosome can be positively correlated with chromosome length. The average unique primer density is 5.73 per Kb of DNA. The highest density occurs on chromosome 1 (6.39 per Kb), and the lowest on chromosome 2 (3.81 per Kb). The density of unique primers in different genomic regions varies, and follows, in descending order: from 0.5 Kb upstream of to transcription start sites (TSS_up_0.5Kb), from transcription end sites to 0.5 Kb downstream of them (TES_down_0.5Kb), code determining sequences (CDSs), introns, 5′-untranslated regions (UTRs), 3′-UTRs, and intergenic regions.

**Table 1:** Distribution of e-PCR primers and polymorphic indels in different regions of the maize genome

Genome region	Total^a^	Unique Primer^b^			Indel^c^ (PIC > 0)			High polymorphic indel (PIC ≥ 0.5)		
		Count	Density^d^ (Kb)	Rate^e^ (%)	Count	Density (Kb)	Rate^f^ (%)	Count	Density (Kb)	Rate^g^ (%)
TSS_up_0.5Kb	978,913	519935	26.27	53.11	135326	6.84	27.76	20419	1.03	4.19
5’-UTR	614,397	352736	11.62	57.41	88155	2.90	25.86	13294	0.44	3.90
3'-UTR	614,453	352784	11.61	57.41	88166	2.90	25.86	13297	0.44	3.90
CDS	2,204,900	1025151	16.29	46.49	77294	1.23	7.89	7272	0.12	0.74
Intron	5,081,799	2050762	11.99	40.36	389605	2.28	19.60	54527	0.32	2.74
TES_down_0.5Kb	967,784	488116	24.66	50.44	116096	5.87	25.17	16589	0.84	3.60
Intergenic	93,492,302	7569844	4.08	8.10	1206314	0.65	18.56	157841	0.08	2.43
Total^h^	102,929,122	11807240	5.74	11.47	1973746	0.96	18.68	264214	0.13	2.50

^a^All primers on the whole 10 chromosomes

^b^Primers were located in unique genomic region

^c^Polymorphism primers were primers with length information in 20 or more than 20 genomes and PIC Value >0

^d^Density was calculated by number/Kb

^e^This rate was the percentage of unique primer against overall primer

^f^This rate was the percentage of unique primer with polymorphisms against unique primer

^g^This rate was the percentage of unique primer with polymorphisms greater than or equal to 0.5 against unique primer

^h^There were 102,929,122 primers in total, and the same primer might be divided into different regions and double counting due to the alternative splicing occurring in maize genome

### Indel variation in the maize genome

The NGS sequencing data from 344 maize genomes consisted of 22,920,398,978 reads, with an average length of 92.78 bp, and an average sequencing depth of 3.02×. The sequencing depth of read qi410 was the lowest, at 0.07×, whereas W64A had the highest sequencing depth, that of 41.46× (Additional file: 1). Read 478 had the most e-PCR hits, that of 8,807,473, accounting for 74.59 % of the total unique primers, whereas qi410 had the fewest hits, 167,605, accounting for 1.42 % of unique primers. There were a sum total of 3,168,631.39 e-PCR hits, accounting for 26.84 % of the unique primers (Additional file: 2). We were able to locate 89.48 % of the unique primers (10,565,398) on over 20 genomes. A total of 1,973,746 indels were identified, a rate of 18.68 % of the unique primers. Chromosome 1 contains the maximum number of indels (330,526), whereas chromosome 10 has the minimum (141,538). The highest indel density occurs on chromosome 1, at 1.09 per Kb; whereas the lowest is on chromosome 2, at 0.62 per Kb; average indel density is 958.79 indels/Mb. The density of indels in various genomic regions follows, from highest to lowest: TSS_up_0.5Kb, TES_down_0.5Kb, 5′-UTR, 3′-UTR, intron, CDSs, and intergenic regions. Accordingly, 38.88 % of the indels are located in genic regions, while 61.12 % are located in intergenic regions.

**Fig. 1:**
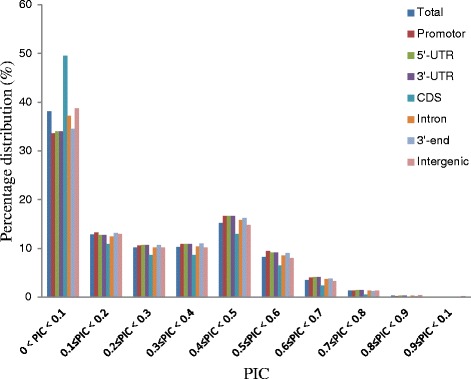
Indel PIC value distribution in different genomic regions

The rate of indel polymorphism varies by genomic region and is listed, from highest to lowest, as follows: TSS_up_0.5Kb, 5′-UTR, 3′-UTR, TES_down_0.5Kb, introns, intergenic regions, and CDSs. Indels in CDS regions not only have a low rate of polymorphism, but also have lower polymorphism information content (PIC) values; 49.52 % of indels within CDSs have PIC values lower than 0.1 (Fig. 1).

### Frequency and distribution of indels in different genomic regions

**Fig. 2:**
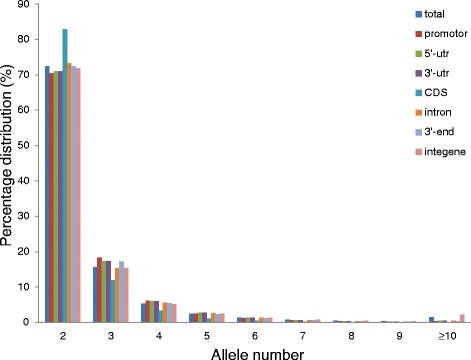
Indel allele number distribution for different genomic regions

**Fig. 3:**
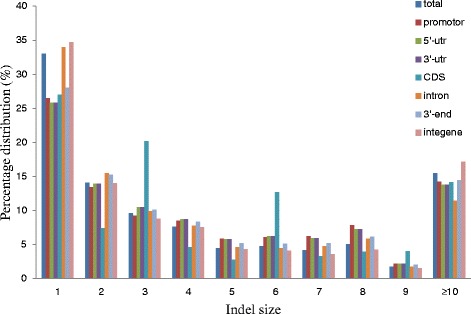
Indel size distribution in different genomic regions

The number of indels decreased as the PIC value increased, and the PIC value of 751,925 indels ranged from 0 to 0.1, accounting for 38.10 % of all indels. There were 264,214 indels with PIC values greater than or equal to 0.5, accounting for 13.39 % of all indels. The number of polymorphic allelic indels ranged from 2 to 107, with an average of 2.76; however, most of the polymorphic indels only had two or three alleles, 72.49 % and 15.63 %, respectively. Proportionally, the number of alleles is nearly the same in different genomic regions, although, CDS regions have a higher proportion of two allele polymorphisms than do other regions, accounting for 82.81 % of all two allele indel polymorphisms (Fig. 2). The size difference between the smallest and largest alleles varies from 1 to 211 bp in length, with the number decreasing as indel size increases. Indels of 1 bp in length account for 33 % of all indels, and indels of 2 bp length account for 14.08 %, while sizes smaller than 11 bp account for 84.53 %. Other than within CDS regions, the proportion of indels in other regions all reduces with an increase in the difference in length between polymorphic indel alleles (Fig. 3).

### Primer design for highly polymorphic indel markers with large major allele differences

Primer pairs for 83,481 unique indels, all with PIC values greater than or equal to 0.5, and with major allele differences greater than or equal to 3 bp, were designed from the maize B73 genome. These indel loci primers were designed to generate PCR products with lengths of 60–100 bp, sizes that polyacrylamide gels can resolve (Additional file: 3). Primer pairs were also designed for 29,403 unique indels, with PIC values greater than or equal to 0.5, and with major allele differences greater than or equal to 8 bp. These indel loci primers were designed with PCR product lengths of 150–300 bp, sizes that agarose gels can resolve (Additional file: 4). The exact positions of these indel markers in the maize genome, as well as the primer sequences, amplicon lengths, PIC values, major allele differences, number of alleles, and number of e-PCR products in the maize genomes evaluated are presented in Additional files: 3 and 4. This data should prove useful in furthering maize genetic research by facilitating primer design in sequences with indels.

### Experimental validation of indel accuracy and polymorphism

**Fig. 4:**
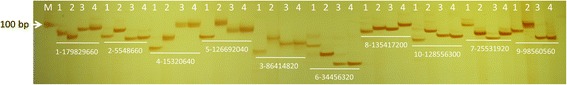
Indel accuracy experimental validation. PCR products from lines 1 through 4 (1212, B73, Mo17, and Zheng58); The numbers below the horizontal line indicate the chromosome and locus of the indel. M: Marker DL2000

Indel accuracy was experimentally validated between the maize 1212 genome and the B73, Mo17, and Zheng58 genomes. With genomic DNA from 1212, B73, Mo17, and Zheng58 as templates, 100 indel loci were PCR-amplified. Of the 100 indel loci, 98 were readily amplified, and 89 of these indel loci were polymorphic between the 1212 and B73 genomes, with an accuracy of 90.82 %. Indel accuracy between 1212 and Mo17 was 90.90 %, while that between 1212 and Zheng58 was 89.80 % (Fig. 4).

**Fig. 5:**
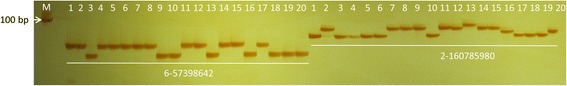
Indel polymorphisms experimental validation. PCR products from lines 1 to 20 are Y0921, JD7275, RP1282, LH8012, 5220-2, 2054, 9HT1736, F19, Sn811, QA356, SC17931, 9HT1736, 9LB050, JD7275, liao1478, mian04185, SCML103, H1277, Qi31912, and Y1035; The numbers below the horizontal line indicate the chromosome and locus of the indel. M: Marker DL2000

Indel polymorphisms were also experimentally validated. We selected another 100 indel loci for PCR amplification. The PIC value of these indel loci in 345 maize genomes ranged from 0.50 to 0.80, with an average PIC value of 0.55, and the allele number ranged from 2 to 13, with an average of 3.50. The PIC value of the indel loci in 20 maize inbred lines ranged from 0.19 to 0.74, with an average of 0.54, and the allele number ranged from 2 to 5, with an average of 2.83 (Fig. 5, Additional file: 5).

## Discussion

Next-generation sequencing technology can produce a huge quantity of DNA sequence data, which becomes a powerful tool for the discovery of high-density molecular markers. A large variety of indel identification software with the main objectives of optimal performance, sensitivity, and specificity is rapidly becoming available [27]. The e-PCR strategy we used identifies indels by aligning flanking sequences to indels rather than mapping complete reads to reference sequences containing indels. This can effectively reduce the influence of indels on alignment. The results of this study show that indels can be efficiently and accurately identified using e-PCR *in silico* assays, which also save considerable time and laboratory costs over using traditional *in vivo*/*vitro* approaches.

The development of high-density molecular markers significantly increases the efficiency of map-based cloning and marker-assisted selection. In this study high-density indel markers, widely distributed across the maize genome, were developed at an average density of one indel per 0.96 Kb, significantly higher than the 0.01 per Kb density developed by Lai et al. [19] in their maize study, and the 0.14 per Kb density used by Mills et al. in their human genome study [1]. Our indel loci reside largely within intergenic regions (1,206,314, 61.12 % of the total), compared with genic regions (767,432, 38.88 %) (Table 1). Indel markers within genes are genic or functional markers [28]. Functional markers are superior to random DNA markers, such as restriction fragment length polymorphism (RFLP), simple sequence repeat (SSR), and amplified fragment length polymorphism (AFLP), because functional markers are completely linked with trait locus alleles [29]. Polymorphism among homologous indels is the basis for developing indel markers. Our experimental results show that the average PIC value and allele number of *in vitro* experimentally developed indel primers are both lower than the *in silico* primers used in e-PCR. This is primarily caused by the difference in resolution between agarose/polyacrylamide gels and e-PCR. We also note that the use of short amplicons reduces the opportunity for the formation of secondary structure, and minimizes length-dependent differential amplification. PCR products were effectively separated and easily scored after 30 min of electrophoresis on 6 % polyacrylamide gels owing to our use of short amplicons and indel to amplicon size ratios larger than 3 %.

The average rate of indel polymorphism is 18.68 % across the maize genome, with TSS_up_0.5Kb regions having the highest polymorphism rate, whereas the CDS regions have the lowest. Intriguingly, polymorphism levels in genic regions of the maize genome were higher than that in intergenic regions. This may be caused by differences in complexity between genic and intergenic regions, as intergenic regions in the maize genome are very rich in repeat sequences. This makes sequence analysis very difficult. The majority of indels in CDS regions had lengths divisible by three, which is a direct result of selection against frameshift mutations (Fig. 3).

The accuracy and identification of unique loci are critically important for developing molecular markers. The predominant error associated with Illumina NGS platforms are substitution errors, which have relatively little effect on indel identification [30]. PCR-based experimental validation shows that indel markers have an accuracy of approximately 90 %. 97.05 % of 20 maize inbred lines contained PCR products for those primers we synthesized for polymorphism validation, which also showed that these indel markers were highly effective. Those PCR products inconsistent with e-PCR results may occur due to non-specific amplification. An increase in read depth per locus can be used to improve the accuracy of indel identification. Moreover, alignment parameters can be set to reduce non-specific amplification by increasing the number of mismatches in the identification of unique loci in the genome, and accuracy can be improved by decreasing the number of mismatches in the analysis of variation between populations. Gel electrophoresis results show that the actual PCR products of indel markers have fewer non-specific bands, which suggests that e-PCR can be a powerful tool for reducing non-specific amplification.

## Conclusions

Here we report a large-scale analysis of genome-wide indel polymorphisms among maize populations, including inbred lines from different stages of breeding history and landraces. Most of these populations are the parents of the commercial hybrid and key lines in today’s global germplasm pool. The indel markers developed in this study provide a simple and efficient tool for any laboratory focusing on map-based gene cloning and molecular marker assisted breeding in maize.

## Methods

### Maize genome sequence sources and plant materials

The genome sequences for B73 (Release ZmB73_RefGen_v2) and Mo17 (454 pyrosequencing data) were downloaded from http://ftp.maizesequence.org/release-5b/assembly/ and http://www.phytozome.net/maize.php, respectively. The 5′-UTR, CDS, 3′-UTR, exon, intron, and intergenic regions were determined based on database annotation (ZmB73_5b_FGS, http://ftp.maizesequence.org/release-5b/filtered-set/). Our TSS_up_0.5 Kb regions are defined as those sequences from 0.5 kp upstream of to transcription start sites, and TES_down_0.5Kb regions are defined as those sequences from transcription end sites to 0.5 kp downstream of them. The re-sequencing data of 344 maize samples were downloaded from National Center for Biotechnology Information (NCBI) (http://www.ncbi.nlm.nih.gov/sra?term=SRA049859 and http://www.ncbi.nlm.nih.gov/sra?term=SRA051245). We determined the genotype of the Chinese waxy maize inbred line 1212, at approximately 10-fold coverage, using Illumina HiSeq 2000 genome sequencing technology.

Twenty-four maize inbred lines were used to test for indel primer accuracy and to validate indel polymorphism levels. This included three elite maize inbred lines (B73, Mo17, and Zheng58), one Chinese waxy maize inbred line (1212), and twenty new maize inbred lines (Y0921, JD7275, RP1282, LH8012, 5220-2, 2054, 9HT1736, F19, Sn811, QA356, SC17931, 9HT1736, 9LB050, JD7275, liao1478, mian04185, SCML103, H1277, Qi31912, and Y1035).

### e-PCR primer design and unique locus primer identification

**Fig. 6:**
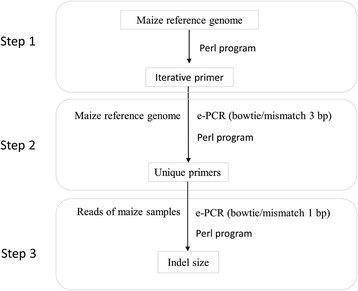
Indel marker development pipeline

**Fig. 7:**
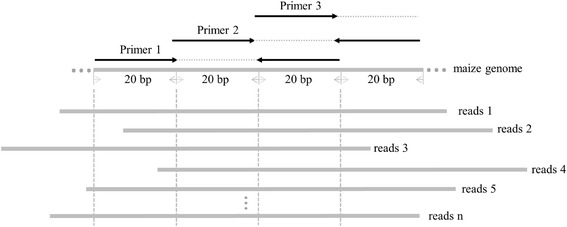
Iterative primer design in maize genome and e-PCR. e-PCR results are available from reads 1, 3, 5, and n

The development of indel markers consists of three steps (Fig. 6). The first step is e-PCR primer design. Using the maize B73 reference genome as a template, 20 bp sequences extracted via a Perl script were used as upstream primers for e-PCR, and after intervals of 20 bp, the reverse complements of another 20-bp sequence were used as downstream primers. The next pair of primers was designed the same way, but 20 bp away from the beginning of the previously designed primer, so that the primers covered the entire genome (step 1 in Fig. 6; Fig. 7). The second step removes redundant/ambiguous primers. All primer sequences were then aligned to the maize B73 reference genome using Bowtie software, an ultrafast and memory efficient tool for aligning short DNA sequences to large sequence databases [31]. Default parameter values were used, except for the number of allowable mismatches between primer and genomic sequences (we used n = 3). Those primers that mapped to multiple positions were eliminated (step 2 in Fig. 6).

### Indel variation among maize populations

The third step is to align all of the valid primer sequences identified in the previous step to short sample reads, and to estimate the length of the resultant e-PCR products (Fig. 6, step 2). The NGSQC toolkit (v.2.3.3) was first used to filter all of the raw data for high-quality reads using a quality score of 20 and above for our cut-off [32]. Unique primer sequences were then aligned against filtered reads from 344 maize sample template sequences via Bowtie, using default parameters, except only allowing one mismatch (step 3 in Fig. 6; Fig. 7). The amplicon length of each e-PCR primer was extracted using a Perl script, and the most frequent length was selected when there were data of various lengths. The allelic diversity of each indel locus that had length information results from ≥ 20 genomes was assessed using the PIC value, which was defined as PIC_*i*_ = 1−∑_*j* = 1_^*n*^*p*_*ij*_^2^, where p_*ij*_ is the frequency of the *j*th pattern for the *i*th marker [33].

### PCR primer design

Unique indel loci were selected for PCR-based primer design. Sequences of 100 bp, including a 20-bp variation region and two 40-bp flanking sequences on each side of the locus were used to automatically design primers with Primer3 [34]. The following parameters were employed: a primer length range from 20 nt to 28 nt, with a 23-nt optimum; a thermal melting temperature (Tm) of 60 °C to 65 °C, with an optimum temperature of 63 °C, and primer pairs must have similar Tm values; a GC content of around 50 %, ranging from 30 to 70 %; and an expected product size of 60 to 90 bp ending with G- or C-rich region at the 3′-end.

### Experimental validation

One hundred pairs of primers evenly distributed on maize chromosomes, all of which had 3–10-bp size differences simultaneously in the genomes of maize inbred lines between 1212 and B73, Mo17, and Zheng58, were selected randomly. Then, DNA from 1212, B73, Mo17, and Zheng58 were used as the template for PCR amplification to validate the accuracy of the indel primer design. One hundred pairs of primers (with PIC values ≥ 0.45 and major allele differences ≥ 3 bp) were selected, and DNA from twenty new maize inbred lines was used as the template for indel polymorphism validation. Genomic DNA was extracted from 2-week-old seedlings using a modified CTAB (cetyltrimethylammonium bromide) DNA extraction protocol [35]. PCR was performed in a reaction mixture of 15 μL, containing 50 ng of total genomic DNA as a template, 1.5 μL 10× buffer (Mg^2+^), 2.0 μL dNTP (2.5 mM), 100 nM of each primer, 2 U Taq polymerase, and ddH_2_O. A C1000 thermal cycler (Bio-Rad, Inc., Hercules, CA) was used for the amplification with the following protocol: an initial denaturation for 3 min at 95 °C, 35 cycles of denaturation for 30 s at 95 °C, annealing for 90 s at 55 °C, with an extension for 90 s at 72 °C; and a final extension for 10 min at 72 °C. PCR products were electrophoresed on a 6.0 % polyacrylamide gel. The PIC value for each marker was calculated using the formula previously described.

## Additional files

Additional file 1:
**List of inbred lines sequenced.** (XLSX 39 kb) Additional file 2:
**Genome differences between varieties and B73.** (XLSX 37 kb) Additional file 3:
**Polymorphic indel markers (3 bp).** The indel markers had PIC values ≥ 0.5, and major allele differences ≥ 3 bp. (XLSX 17940 kb) Additional file 4:
**Polymorphic indel markers (8 bp).** The indel markers had PIC values ≥ 0.5, and major allele differences ≥ 8 bp. (XLSX 6664 kb) Additional file 5:
**Indel marker experimental validation.** (XLSX 16 kb)
